# Agreement of axial T2-MRI versus Contrast-Enhanced CT for solid renal mass active surveillance

**DOI:** 10.1007/s00261-026-05376-6

**Published:** 2026-03-24

**Authors:** Bendik Breen, Khalefa Alghofaily, Heidi Søgaard Christensen, Jens Brøndum Frøkjær, Ole Graumann, Mats Kleivane, Lise Medrud, Rasa Mikalone, Stig Müller, Gintare Naujokaite, Anne Negård, Ivar Mjåland Salte, Nicola Schieda, Jens Borgbjerg

**Affiliations:** 1 Department of Radiology, Akershus University Hospital, Lillestrøm, Norway; 2 Institute of Clinical Medicine, University of Oslo, Oslo, Norway; 3 Department of Radiology, Ottawa Hospital, Ottawa, Canada; 4 Department of Radiology, College of Medicine, Qassim University, Qassim, Saudi Arabia; 5 Department of Clinical Medicine, Aalborg University, Aalborg, Denmark; 6 Department of Radiology, Aalborg University Hospital, Aalborg, Denmark; 7 Department of Radiology, Aarhus University Hospital, Aarhus, Denmark; 8 Department of Radiology, Regionshospitalet Viborg, Viborg, Denmark; 9 Department of Urology, Akershus University Hospital, Lillestrøm, Norway

**Keywords:** Small renal masses, Active surveillance, Non-contrast MRI, Contrast-enhanced CT, Tumor diameter measurement

## Abstract

**Purpose:**

Active surveillance (AS) of small renal masses (SRMs) typically relies on contrast-enhanced CT for serial size measurement. Non-contrast (NC) MRI protocols may provide an alternative. To assess the agreement of maximum axial tumor diameter and tumor nearness between NC MRI and contrast-enhanced CT for SRM surveillance, and to compare diagnostic confidence in tumor delineation across modalities.

**Methods:**

We retrospectively identified 50 patients (mean age, 62.9 years; 44% women) who underwent both contrast-enhanced CT and axial T2-weighted MRI within 3 months as part of AS protocol. Nine radiologists from five hospitals independently assessed maximum tumor diameter (axial plane) and tumor nearness to the collecting system (TN, three-tier scale) and subjectively graded their diagnostic confidence in delineating the tumor contour (DC, 5-point scale) in 50 CT and MRI scans across four randomized reading sessions. Observer agreement for diameter assessment was quantified using the limits of agreement with the mean (LOAM), whereas TN and DC were analyzed using Gwet’s AC2.

**Results:**

Mean tumor diameters were 19.9 mm for CT and 19.5 mm for MRI (mean difference, 0.4 mm; *p* = 0.051). Reproducibility LOAMs were ± 2.9 [2.7–3.2] mm (CT) and ± 3.1 [2.9–3.4] mm (MRI), with overlapping confidence intervals. TN ratings and agreement were similar for CT and MRI (*p* = 0.61; *p* = 0.86, respectively), whereas DC ratings were higher and interobserver agreement was better for CT (*p* < 0.001 and *p* = 0.009, respectively)..

**Conclusion:**

NC axial T2-weighted MRI demonstrated tumor diameter agreement comparable to contrast-enhanced CT, along with comparable TN ratings and agreement, whereas CT yielded higher DC and better interobserver agreement for DC.

**Supplementary Information:**

The online version contains supplementary material available at https://doi.org/10.1007/s00261-026-05376-6.

## Introduction

Active surveillance (AS) has emerged as a viable and guideline-endorsed management strategy for small renal masses (SRMs), defined as solid tumors ≤ 4 cm (clinical stage T1a). This approach aims to avoid overtreatment and preserve renal function, particularly relevant given the increasing incidence of incidentally detected renal masses [1]. International guidelines from leading organizations, including the American Urological Association (AUA) [2], European Association of Urology (EAU) [3], and National Comprehensive Cancer Network (NCCN) [4], now consistently recommend AS as an acceptable initial strategy for carefully selected patients with SRMs, particularly emphasizing that tumor size is a key predictor of aggressiveness, with smaller tumors - especially those under 2 cm - demonstrating significantly lower risks of adverse pathology and metastatic potential [5]. Indeed, as tumor size increases, so does the likelihood of aggressive histological features [6]. Furthermore, recent studies suggest acceptable oncological outcomes of AS even among younger patient populations (< 60 years old), further broadening the applicability and acceptance of conservative management strategies [7].

Current imaging modalities are not sufficiently reliable for definitively distinguishing benign from malignant SRMs due to overlapping radiographic features [8]. Hence, the primary metric for prompting intervention (such as surgery or ablation) during AS, according to guidelines, is imaging-based tumor size and growth rate, typically defined as growth exceeding 5 mm per year or reaching an absolute size of 4 cm [5].

Contrast-enhanced CT remains the standard imaging modality for surveillance of SRMs due to its widespread availability, rapid imaging acquisition, and high spatial resolution [9]. However, repeated CT scans throughout prolonged surveillance periods raise significant concerns related to cumulative radiation exposure, particularly relevant for younger patients with long life expectancy and potentially decades of surveillance ahead [10, 11]. Moreover, despite its diagnostic strengths, CT with intravenous contrast can be problematic for patients with impaired renal function, with risks of contrast-associated acute kidney impairment [12].

Alternative imaging modalities, including ultrasound and magnetic resonance imaging (MRI), have been considered for AS protocols. However, ultrasound, despite being radiation-free and cost-effective, is hampered by substantial inter- and intra-observer variability, limiting its reliability for long-term tumor size monitoring [9, 13]. Conversely, traditional MRI with intravenous contrast, although radiation-free and superior in soft-tissue contrast, is costly, time-consuming, and logistically challenging in clinical practice.

To address these challenges, non-contrast (NC) MRI protocols, primarily consisting of axial T2-weighted sequences, have been suggested as a feasible alternative for surveillance [14–16]. Establishing abbreviated NC-MRI as a robust surveillance alternative, relying on a T2-weighted MRI sequence could significantly reduce radiation exposure compared to CT, lessen the use of contrast media, shorten MRI examination time, and improve both accessibility and patient adherence. To date, only one prior study has investigated NC-MRI for AS of SRMs [17], highlighting the need for further research to clarify the role of NC-MRI in AS protocols.

This multi-observer study aimed to evaluate agreement comparing NC MRI and routine-dose contrast-enhanced CT in the surveillance of small renal masses.

## Materials and methods

The study was approved by the Norwegian Regional Committees for Medical and Health Research Ethics (reference 285666) and by the institutional data protection officer with a waiver for informed patient consent.

### Patients

We queried the Picture Archiving and Communication System (PACS) at Akershus University Hospital to identify adult patients (≥ 18 years) undergoing AS for a solid SRM (≥ 25% of the mass composed of enhancing tissue) [18] between January 2010 and December 2022. Patients were required to have undergone a contrast-enhanced CT performed according to our institutional renal mass CT protocol, and an MRI had to have been performed within 3 months of this CT, either for AS or other clinical purposes with an axial T2-weighted sequence.

Exclusion criteria included infiltrative and non-circumscribed masses, predominantly cystic masses with less than 25% enhancing tissue, masses containing macroscopic fat, masses measuring less than 1 cm or greater than 4 cm, severe respiratory artifacts on either modality, no axial T2-sequence taken as part of the MRI exam, and NC CT examinations.

**Fig. 1:**
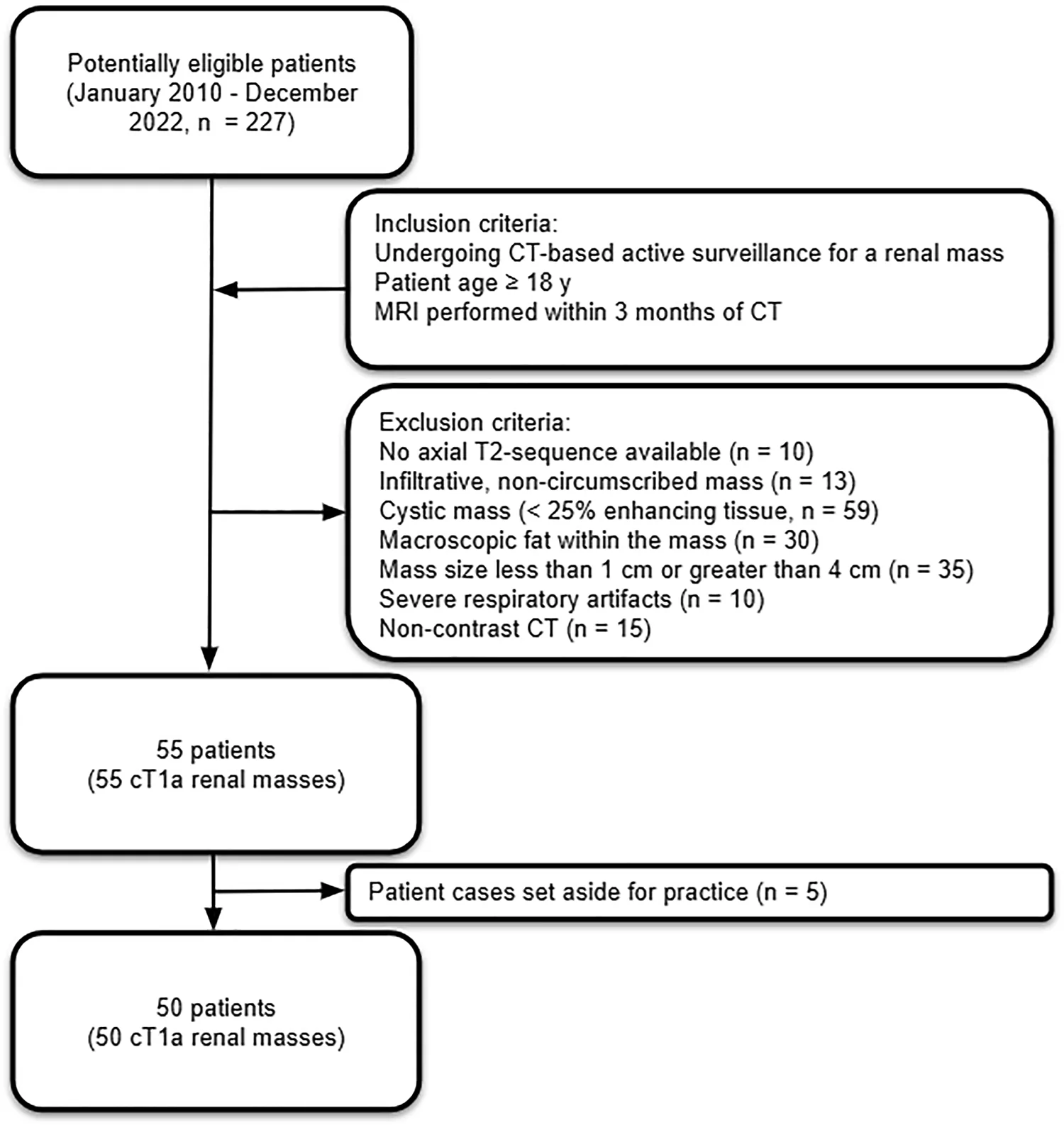
Study flow diagram

If the CT scan was deemed acceptable regarding infiltration and tumor circumscription, the case was included regardless of the MRI findings concerning these parameters. Figure 1 shows the flow of patient selection. Patient eligibility was evaluated by an abdominal radiologist (JB) with 12 years of experience with CT.

### CT and MRI protocols

**Fig. 2:**
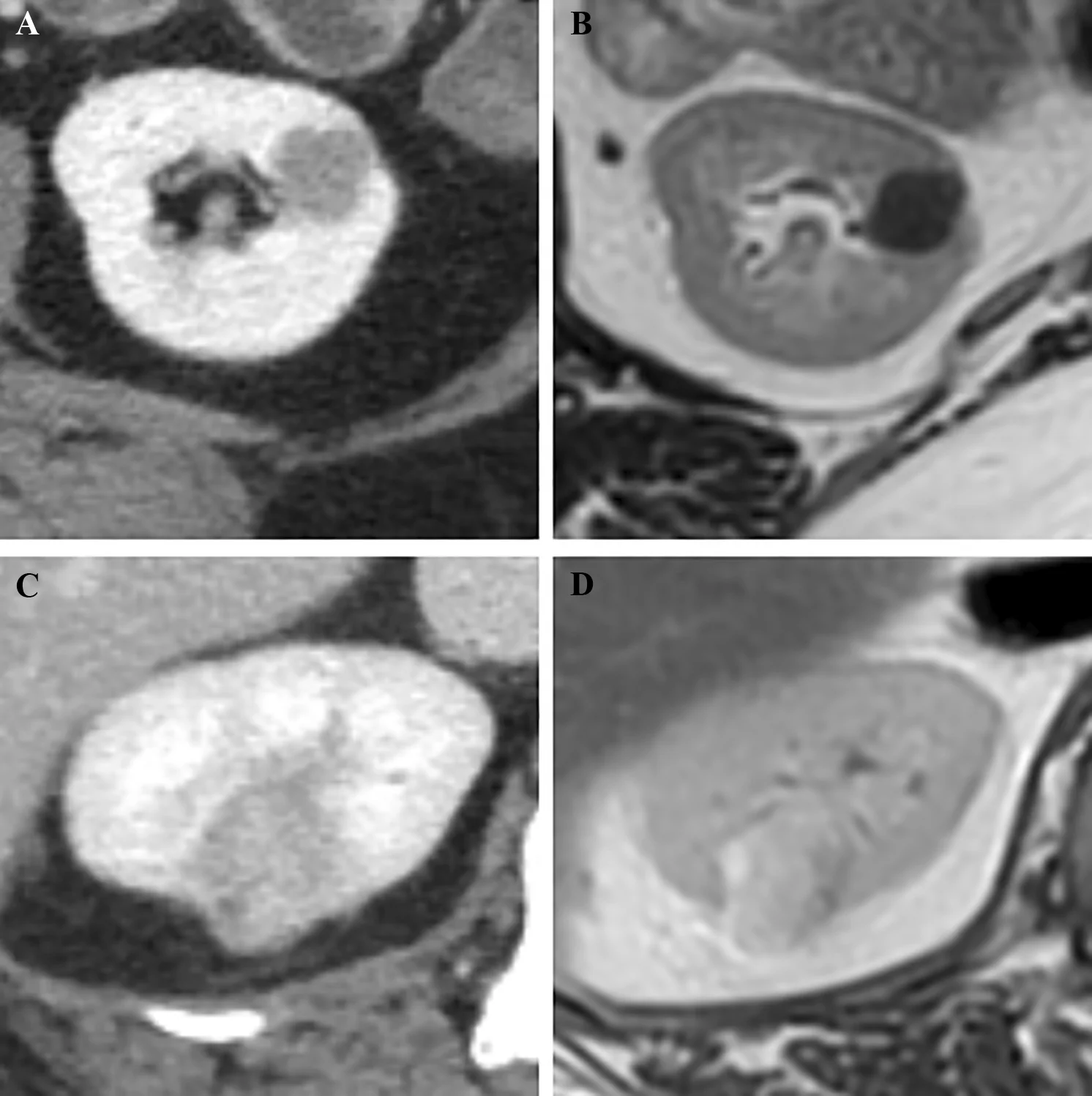
Presents contrast-enhanced CT and T2-weighted images in the axial plane of two small renal masses. A 1.7 cm right-sided, well-circumscribed, endophytic small renal mass in a 70 year-old female patient depicted as homogenous and with a lower degree of enhancement compared to renal parenchyma on contrast-enhanced CT (**A**) and lower signal intensity relative to renal parenchyma on an MRI T2-sequence (**B**). A 2.7 cm right-sided, partly exophytic small renal mass in a 65-year-old male patient depicted as slightly heterogeneous with contrast-enhanced CT (**C**) and with hyperintense signal intensity relative to renal parenchyma on a T2-weighted sequence (**D**)

The CT protocol consisted of contrast-enhanced upper abdominal CT scans performed on one of five different Philips CT systems (Philips Healthcare, The Netherlands), with detector configurations ranging from 64 to 256 slices. A bodyweight-adapted volume of Iohexol 350 mg I/mL (Omnipaque 350, GE Healthcare) was administered at a dosage of 2 mL/kg, with an injection duration of 35 s and a scan delay of 80 s. Scans were acquired with a tube voltage of 120 kV, matrix size of 512 × 512, and automatic tube current modulation (DoseRight 3D-DOM) enabled, with the DoseRight Index set between 19 and 22. Images were reconstructed using the hybrid iterative reconstruction algorithm iDose⁴ (level 2 to 5) with a soft tissue filter (Filter B). Reconstructed axial image slice thickness varied between 0.9 and 3 mm, depending on the specific scanner and clinical protocol (Fig. 2).

MRI examinations were performed on seven different 1.5 and 3 Tesla clinical MRI systems (Philips and Siemens Healthcare). Both conventional multi-shot turbo spin echo (TSE) T2-weighted sequences and single-shot T2-weighted acquisitions (HASTE on Siemens systems and SS-TSE on Philips systems) were used. All T2-weighted sequences were acquired without fat suppression, and most employed partial Fourier techniques. Depending on the clinical and anatomical context, images were acquired either with respiratory triggering or in a breath-hold. Detailed MRI sequence parameters and acquisition times are summarized in Table 1.

**Table 1:** Ranges of MRI parameters of T2 sequences

TR (ms)	500 to 10,223
TE (ms)	70 to 260
Flip angle (°)	90 to 160
Slice thickness (mm)	3 to 7
Slice spacing (mm)	3 to 8
Image matrix (px)	288 × 288 to 864 × 864
Pixel spacing (mm)	0.37 to 1.24
Signal averages	1 to 2
Acquisition time (s)	35 to 460

Parameters are numbers presented as ranges

### Observers

A total of nine radiologists from four university-affiliated hospitals and one regional hospital in Norway, Denmark, and Canada (Akershus University Hospital, Aarhus University Hospital, Aalborg University Hospital, Regional Hospital of Viborg, and The Ottawa Hospital) participated in the study. All readers had ≥ 4 years of experience in abdominal CT and MRI interpretation, with a mean of 11.3 ± 5.7 years (range, 4–23).

### Observer assessments

**Fig. 3:**
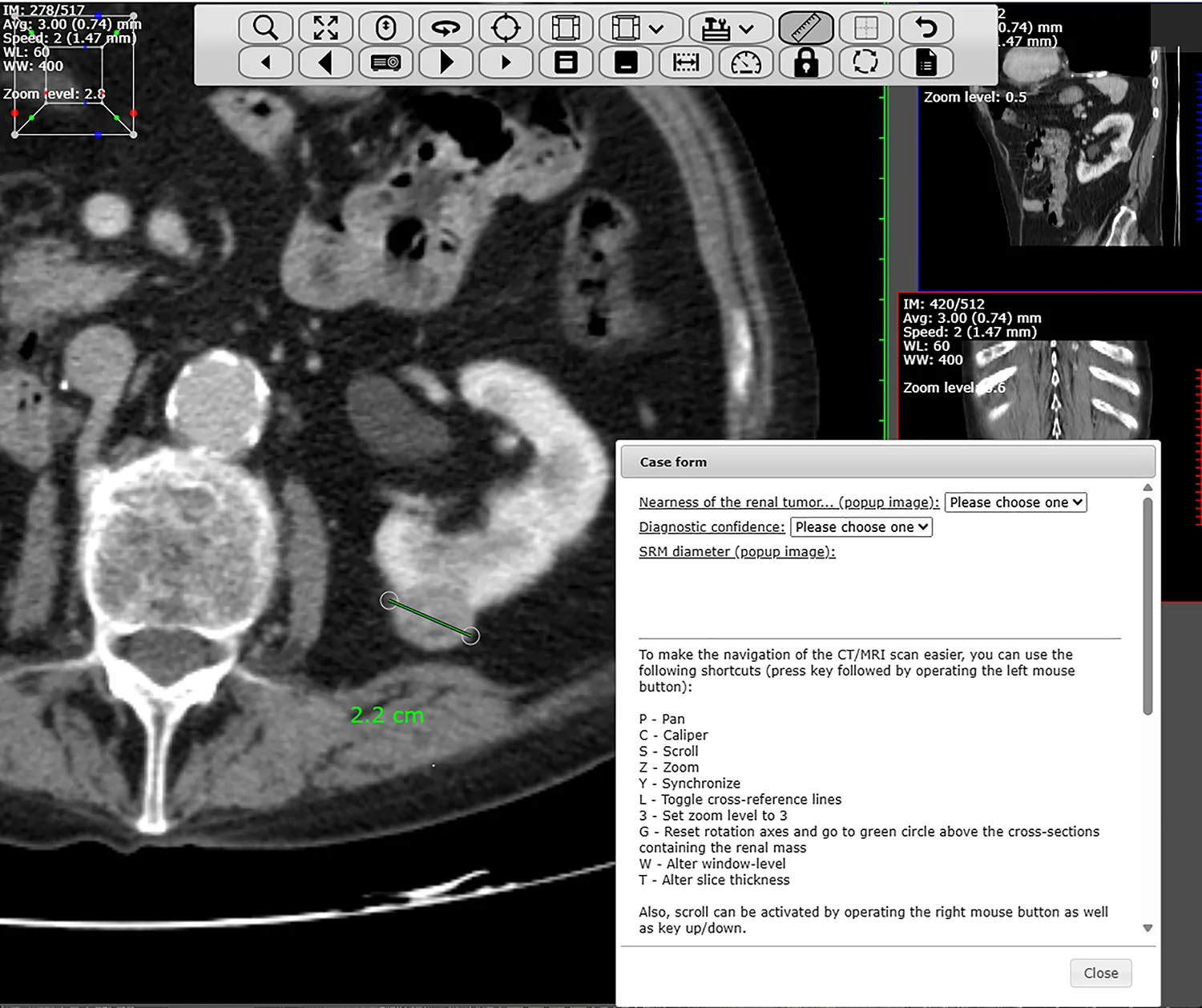
Screenshot of the web-based Digital Imaging and Communications in Medicine viewer equipped with a case report form. A 2.2 cm posterior exophytic small renal mass in the left kidney is depicted on a contrast-enhanced axial CT image accompanied by reconstructions in the coronal and sagittal planes

The paired axial contrast-enhanced CT and axial T2-weighted sequences from each patient were anonymized and uploaded to a previously described web-based platform developed for imaging observer performance studies [19]. This platform supports image review directly through a web browser and automatically captures observer responses. It is integrated with a DICOM viewer that offers multiplanar reconstruction functionality (Fig. 3) [20].

**Fig. 4:**
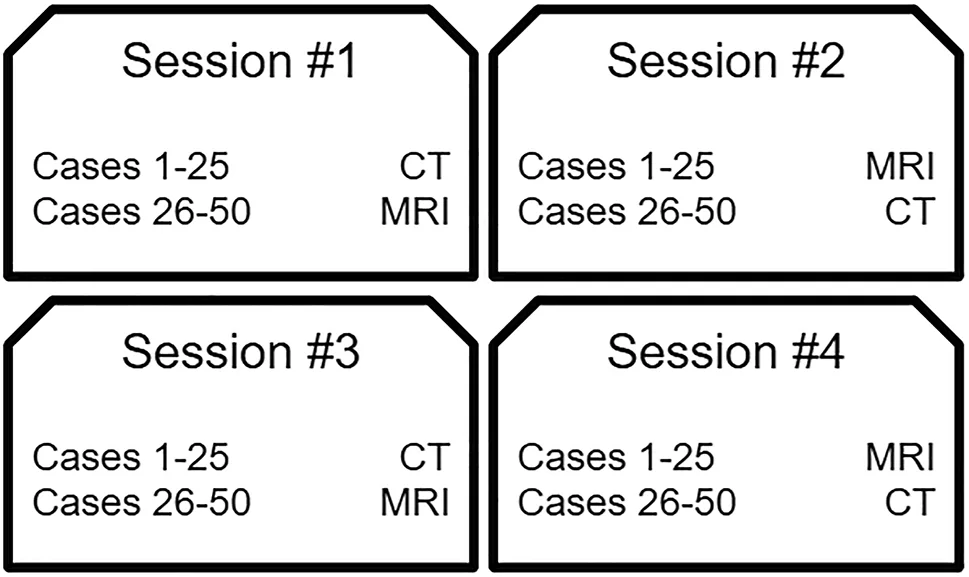
Overview of the reading scheme in a mixed-order setup. Each of the four reading sessions included 50 renal tumors acquired with either CT or MRI, with a minimum two-week washout period between sessions. For each of the nine observers, the order of the first two sessions (Session #1 and Session #2) was randomized. The remaining two sessions were presented in reverse order, such that if an observer initially received Session #2 followed by Session #1, they would later receive Session #4 followed by Session #3. Within each session, the order of scan presentation was randomized

The nine observers independently evaluated the 50 patients’ CT and MRI scans separately, with each scan read twice across four distinct reading sessions to assess intra- and interobserver variability. To mitigate recall bias, a mixed-order reading design was implemented, where each session comprised 50 scans, with a minimum two-week washout period between sessions [21]. The CT and MRI examinations were distributed across the sessions, and each patient’s scan appeared only once per session (Fig. 4). Observers were blinded to the technical parameters of the CT and MRI scans, clinical information, and the assessments made by other observers.

Written instructions and instructional videos on the use of the DICOM viewer were provided. Additionally, observers completed a training session involving five paired CT and MRI cases to familiarize themselves with the viewer interface and the structure of the case report form (Fig. 3).

Observers measured the maximum tumor diameter, with caliper placement restricted to the axial plane in accordance with prior AS protocols where axial measurements are most consistently reported [9]. This approach also reflects the use of axial T2-weighted imaging as a suggested cornerstone of abbreviated MRI protocols for SRM surveillance [14]. To aid in locating the target renal mass, the DICOM viewer displayed a green marker at the z-axis level immediately above the renal mass to be evaluated. In addition, the observers evaluated tumor nearness (TN), an ordinal variable representing the minimum distance from the tumor to the renal collecting system or sinus, categorized into three tiers: ≤4, > 4-<7, and ≥ 7 mm, corresponding to ratings of 3, 2, and 1, respectively. TN is part of the RENAL score, and a well-established predictor of overall complications, postoperative hemorrhage after nephron-sparing surgery, malignancy risk, and adverse pathological features [1, 22, 23]. Observers subjectively graded their diagnostic confidence in delineating the tumor contour (DC) - reflecting tumor–parenchyma contrast, margin clarity, and the impact of noise or artifacts - on a 5-point Likert scale (1 = poor, 2 = fair, 3 = good, 4 = very good, 5 = excellent).

### Statistical analysis

Statistical analyses were conducted using Python (v. 3.10, https://www.python.org). The normality of the distribution of quantitative variables was assessed both visually and with the Shapiro-Wilk test. Data were reported as mean ± standard deviation (SD) or median with interquartile range (IQR), depending on the normality of the distribution.

We used the limits of agreement with the mean (LOAM) formulated by Christensen et al. to assess observer agreement for tumor diameter measurements in a multi-observer setup [24], and extended the method to encompass both reproducibility LOAM (i.e., how much an observer’s measurement may plausibly deviate from the mean of all observers’ measurements on the specific renal mass) as well as repeatability LOAM (i.e., how much a given observer’s measurement may plausibly deviate from the mean of all measurements performed by that particular observer on the specific renal mass) [25].

We aimed to include a sufficient number of observers to obtain 95% confidence interval (CI) width of approximately 0.5 mm for the reproducibility LOAM. A small pilot study was conducted, in which three observers measured the 50 CT and MRI scans. Given the 50 patient cases and based on the observed measurement variability and the method described by Christensen et al. [24], we estimated that nine observers would be required to achieve an expected 95% CI width of 0.5 mm for the 95% reproducibility LOAM.

For SRM diameter, all observer/session measurements were averaged to yield one CT and one MRI value per patient, avoiding pseudoreplication. Paired t-tests were used to compare modalities. For TN and DC, analyses were based on ratings from the first reading session only. For each patient and modality, the median score across all observers was calculated, yielding one paired value per patient. These patient-level medians were compared between CT and MRI using the sign test, which does not assume equal spacing between ordinal categories. Distributions of ratings are reported as counts and percentages for each category. Inter-observer agreement for all nine observers for TN and DC was assessed using Gwet’s AC2 [26], and differences between CT and MRI were evaluated with a non-parametric bootstrap that resampled patients within each modality to obtain the 95% CI and p-value for the AC2 difference.

## Results

### Patient characteristics

A total of 50 patients were included in the study, with each having a contrast enhanced CT and MRI examination. The patients had a mean age of 62.9 (± 13.3, range 39–85) years and 44% of the cohort were female (Table 2).

**Table 2:** Patient demographics, tumor characteristics, and CT radiation exposure data

Patients (*n* = 50)	
Age (years)	62.9 (± 13.3, 39–85)
Sex, n (%)	
Female	22 (44)
Male	28 (56)
Body mass index (kg/m^2^)	27.0 (± 6.0, 17.3–40.4)
Tumor characteristics (*n* = 50)	
Mean tumor diameter (mm)⁋	19.9 (± 7.7, 8.0–43.2)
TN (mm)	
≤ 4	22 (44)
> 4 but < 7	8 (16)
≥ 7	20 (40)
Exophytic-endophytic characteristics	
≥ 50% exophytic	19 (38)
< 50% exophytic:	23 (46)
100% endophytic	8 (16)
Radiation dose	
Mean scan length (cm)*	26.6 (± 4.3, 20.6–47.2)
Mean DLP (mGy x cm)	461.4 (± 234.1, 104.4–1229.1)
Mean CTDIvol (mGy)	17.0 (± 7.4, 4.9–40.6)
Effective dose (mSv)†	6.9 (± 3.5, 1.6–18.4)

Data are numbers with percentages in parentheses or means ±SDs with ranges in parentheses. Body mass index is calculated as weight in kilograms divided by height in meters squared. The mean tumor diameter, and tumor nearness to the collecting system or sinus (TN) are based on the CT dataset, which means reflecting the combined assessments of all nine observers

*CTDIvol* volume CT dose index, *DLP* dose length product

⁋ All renal masses met the ≤4 cm size criterion according to the selecting radiologist’s clinical measurement. One lesion showed a slightly larger diameter when re-measured by multiple blinded observers, representing ordinary interobserver variability rather than incorrect case selection

* From the diaphragm to the iliac crest

† Effective dose = DLP × abdominal weighting factor (0.015 mSv · mGy^−1^ · cm^−1^)

### Observer agreement

**Fig. 5:**
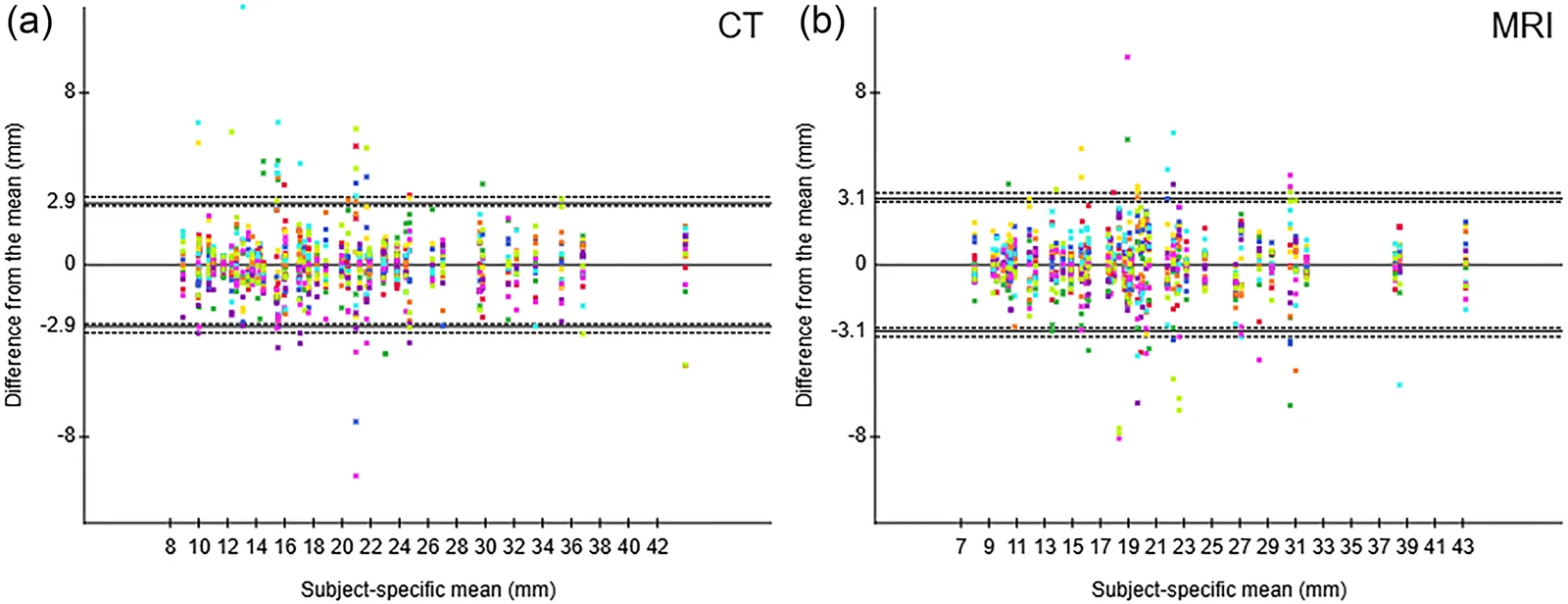
Observer agreement plots for renal mass diameter measurements (in millimeters) across 50 patients imaged with contrast-enhanced CT (**a**) and an MRI T2-sequence (**b**). The horizontal axis represents the subject-specific (patient) mean renal mass diameter measurements, while the vertical axis shows the difference between individual observer measurements and the subject-specific mean. Each colored dot represents an individual measurement by one of the nine observers. Note that some dots are superimposed due to overlapping data points. Horizontal solid lines denote the upper and lower 95% reproducibility limits of agreement with the mean, along with a line of zero difference. Dashed lines show the 95% confidence intervals (CIs) for the limits of agreement with the mean. Reproducibility LOAMs were ± 2.9 mm (95% CI, ± 2.4 to ± 3.4 mm) for CT and ± 3.1 mm (95% CI, ± 2.6 to ± 3.6 mm) for MRI, with overlapping CIs indicating comparable reproducibility between modalities

Across the four reading sessions, the nine observers performed a total of 1800 patient scan-assessments, encompassing repeated assessments of both CT and MRI scans. For CT and MRI individually and across reading sessions, the mean, range, and 95% limits of agreement with the mean, along with the corresponding variance components, are presented in Table 3. Agreement plots are shown in Fig. 5.

**Table 3:** Mean renal mass diameter and 95% LOAM including variance components (all in mm) of renal mass measurements by nine observers (200 measurements each) for the contrast-enhanced CT and MRI T2-sequence datasets

Metric	CT	MRI
Mean (95% CI)	19.9 (17.7 to 22.1)	19.5 (17.2 to 21.7)
LOAM_reproducibility_ (95% CI)	± 2.9 (2.7 to 3.2)	± 3.1 (2.9 to 3.4)
LOAM_repeatability_ (95% CI)	± 1.6 (1.5 to 1.7)	± 1.8 (1.7 to 1.9)
σ_A_ (95% CI)	7.9 (7.1 to 8.7)	8.1 (7.3 to 8.9)
σ_B_ (95% CI)	0.4 (0.3 to 0.5)	0.4 (0.3 to 0.5)
σ_R_ (95% CI)	1.5 (1.4 to 1.5)	1.6 (1.5 to 1.7)
σ_AB_ (95% CI)	0.9 (0.8 to 1.0)	0.9 (0.8 to 1.1)
σ_E_ (95% CI)	1.2 (1.1 to 1.2)	1.3 (1.2 to 1.4)

Data in parentheses are 95% CIs. σ_A_ = the inter-subject variance component, σ_B_ = the inter-observer variance component, σ_R_ = the composite residual variance component, σ_AB_ = the subject-observer interaction variance component, σ_E_ = the error variance component, *LOAM* limits of agreement with the mean, *CI* confidence interval

The mean diameter measured across observers was 19.9 (± 7.7) mm for CT versus 19.5 (± 7.9) mm for MRI, resulting in a mean difference of 0.4 mm (95% CI: 0.0 to 0.9, *p* = 0.051) between CT and MRI.

The reproducibility LOAM were comparable for both modalities (± 2.9 [2.7–3.2] vs. ± 3.1 [2.9–3.4] mm for CT and MRI) with overlapping CIs of the LOAM (Fig. 5). A narrower repeatability LOAM for CT was observed compared to reproducibility LOAM for CT and MRI, with widths of ± 1.6 [1.5–1.7] versus ± 1.8 [1.7–1.9] mm, which also had overlapping CIs. Furthermore, the two agreement plots give no indication of heteroscedasticity associated with renal mass diameter.

Tumor nearness and DC metrics, including rating distributions, and interobserver agreement are summarized in Table 4. Briefly, for TN, the sign test showed no significant difference between modalities (*p* = 0.61). The agreement between all nine observers, as measured by Gwet’s AC2, was 0.66 (95% CI 0.55–0.77) for CT and 0.65 (95% CI 0.53–0.77) for MRI. The bootstrap comparison between CT and MRI showed no evidence of a difference (observed difference 0.011, 95% CI − 0.11 to 0.14, *p* = 0.864). For diagnostic confidence, the sign test indicated a statistically significant difference favoring CT (*p* < 0.001). Gwet’s AC2 was 0.46 (95% CI 0.40–0.52) for CT and 0.34 (95% CI 0.28–0.40) for MRI. The bootstrap comparison showed significantly higher agreement for CT (observed difference 0.11, 95% CI 0.04 to 0.19, *p* = 0.009).

**Table 4:** Tumor nearness and diagnostic confidence rating distributions, and Gwet’s AC2 for CT and MRI

Metric	CT	MR	*p*
TN			
Rating distribution, n (%)	≤ 4 mm: 21 (42%) > 4–<7 mm: 4 (8%) ≥ 7 mm: 25 (50%)	≤ 4 mm: 17 (34%) > 4–<7 mm: 6 (12%) ≥ 7 mm: 27 (54%)	*0.61
Gwet’s AC2 (95% CI)	0.66 (0.55–0.77)	0.65 (0.53–0.77)	†0.864
DC			
Rating distribution, n (%)	2: 1 (2%) 3: 20 (40%) 4: 27 (54%) 5: 2 (4%)	2: 8 (16%) 3: 31 (62%) 4: 11 (22%)	*<0.001
Gwet’s AC2 (95% CI)	0.46 (0.40–0.52)	0.34 (0.28–0.40)	†0.009

Gwet’s AC2 coefficients are shown with 95% CI in brackets

*paired sign test (difference in medians) †AC2 difference (interobserver agreement comparison)

TN tumor nearness, *DC* diagnostic confidence, *CI *confidence interval

## Discussion

This study was conducted to evaluate whether NC MRI, using a single T2-weighted sequence, could serve as a radiation-free alternative to contrast-enhanced CT for the longitudinal surveillance of SRMs in patients undergoing AS. Our primary finding was that axial T2-weighted sequence measurements of SRMs were comparable to contrast-enhanced CT when evaluating the maximal tumor diameter in SRMs, as reflected by the reproducibility LOAM.

Regarding TN, we did not find any significant difference in ratings between CT and MRI. Importantly, interobserver agreement for TN was similar for both modalities, supporting that abbreviated MRI can provide sufficient agreement for this clinically relevant parameter in the AS setting.

Diagnostic confidence in delineating tumor margins was higher for CT than MRI, likely reflecting greater tumor–parenchyma conspicuity and greater reader familiarity with CT in SRM surveillance. However, this higher confidence did not translate into superior measurement agreement, as LOAM values were similar across modalities. Diameter reproducibility is driven by caliper placement variability, which appears robust even when margin definition varies slightly. This is consistent with prior work showing that subjective confidence may change before objective performance metrics are affected as observed by Fletcher et al.during CT dose reduction [27].

Our data show a level of agreement in renal mass diameter assessment that is on par with the well-recognized CT study by Punnen et al. [28], where three radiologists evaluated 29 RMs. This work was a central contributor to the establishment of the widely adopted 5 mm/year growth threshold cited in guidelines as a key trigger for considering intervention during AS. Our work also echoes more recent studies by Borgbjerg et al. [29] evaluating lower-dose versus routine-dose contrast-enhanced CT for active surveillance of SRMs, where observer agreement in tumor diameter measurements and assessment of TN remained robust despite objectively lower image quality and decline in diagnostic confidence in delineating tumor contour.

Mawi et al. evaluated NC T2-weighted MRI against contrast-enhanced MRI for SRM surveillance, the only prior study on unenhanced MRI in this setting [17]. They found intraobserver intermodality limits within the 5 mm growth threshold, but interobserver limits exceeded this margin for both sequences [17, 30]. With only 39 patients and two observers, the study provided limited certainty about the agreement estimates and restricted their generalizability. In contrast, our multi-observer study shows reproducibility within 5 mm, providing evidence that abbreviated NC MRI is a reliable alternative to contrast-enhanced CT in AS of SRMs.

An increased number of patients are expected to undergo AS in the future [6] and it is well-recognized that there is a variable latency period between radiation exposure and the development of solid cancers that extends up to a decade or longer in most cases [31]. This latency, combined with the relatively recent emergence of literature on AS for SRMs [32] - particularly in younger patients with a median follow-up of approximately 5 years [33] - raises concerns about the long-term safety of CT-based surveillance in this population. In terms of implications for clinical practice, once a SRM has been deemed solid, our results support the use of a focused MRI protocol for surveillance, providing an intravenous contrast- and radiation-free alternative. In addition, while some authors advocate for using the same imaging modality consistently throughout the surveillance period [9], our findings indicate that alternating contrast-enhanced CT and NC MRI could be feasible in active surveillance, given the comparable agreement in diameter and tumor-nearness assessment. Although consensus is still lacking regarding the optimal combination of imaging modalities and surveillance intervals, our results suggest that a NC MRI obtained for an unrelated clinical indication might serve as an acceptable interim assessment. This could help avoid redundant imaging, radiation exposure, and unnecessary visits. However, these strategies require confirmation in prospective longitudinal studies evaluating within-patient growth trajectories before they can be adopted in routine practice. An additional potential advantage of abbreviated NC MRI has reduced cost compared to a full multiparametric MRI with intravenous contrast, as demonstrated in other abdominal applications such as pancreatic cyst and hepatic fat/iron protocols [34, 35]. A direct economic comparison with contrast-enhanced CT has not yet been performed, and future work will be required to determine whether abbreviated MRI can be cost-competitive in this setting.

Our study has limitations. First, unlike clinical practice, observers evaluated lesions without access to prior examinations, which may underestimate the true level of agreement. Second, we did not assess longitudinal growth, an important endpoint in AS; instead, we focused on inter- and intraobserver agreement in diameter assessment, which is a commonly used surrogate for growth detection in radiological studies. Third, CT and MRI were performed within a 3-month interval, but SRMs typically exhibit slow growth, with reported median growth rates of only 1–2.7 mm/year across prospective active surveillance cohorts [36]. Any interval growth over such a short period would be minimal and likely introduce minor random rather than systematic bias. The near-zero mean CT–MRI diameter difference in our data supports that interval timing did not materially influence the results. Fourth, this study evaluated agreement between CT and NC MRI measurements rather than accuracy against a histopathologic or volumetric ground truth. Fifth, the renal masses were imaged on a variety of MRI systems with differing acquisition parameters, including variations in slice thickness and sequence type. This heterogeneity may have introduced measurement imprecision and represents a potential source of bias. At the same time, it reflects real-world clinical practice where MRI protocols may not be standardized for SRM surveillance, which may enhance the external validity of our findings. Nonetheless, reproducibility estimates should be interpreted with caution in light of this protocol variation. Sixth, the retrospective study design, which relies on patients from a single center may introduce population bias. To mitigate this, we included radiologist observers from five different institutions. Seventh, the renal mass sample size is relatively small, albeit comparable to previous studies investigating agreement of CT-based tumor diameter assessment [28, 37]. Eight, cystic renal masses were excluded, as their surveillance depends on gadolinium-enhanced MRI for Bosniak classification, which prioritizes morphological features over size [38]. Finally, the maximum axial tumor diameter was evaluated to remain consistent with previous research. However, we recognize that this measurement may not fully reflect the complexity of tumors with irregular shapes.

In conclusion, this multi-observer study demonstrated that a single-sequence, NC T2-weighted MRI offers comparable agreement to contrast-enhanced CT in measuring SRM diameter during AS. Agreement for TN was similar across modalities, and while CT yielded higher DC, this did not translate into superior measurement consistency. Our findings support abbreviated NC-MRI as a radiation-free and intravenous contrast–free alternative for surveillance of SRMs.

## Supplementary Information

Below is the link to the electronic supplementary material.

Supplementary Material 1

## Data Availability

The datasets analyzed during the current study are available from the corresponding author on reasonable request.
